# Environmental resources moderate the relationship between social support and school sports participation among adolescents: a cross-sectional analysis

**DOI:** 10.1186/1479-5868-8-34

**Published:** 2011-04-18

**Authors:** Dan J Graham, Margaret Schneider, Sally S Dickerson

**Affiliations:** 1 University of California, Irvine, Department of Psychology and Social Behavior, 4558 Social and Behavioral Sciences, Irvine CA 92697, USA; 2 University of California, Irvine, Department of Planning, Policy, and Design, 258 Social Ecology I, Irvine CA 92697, USA; 3 University of Minnesota, Twin Cities, Division of Epidemiology and Community Health, 1300 S. 2nd Street, Suite 300, Minneapolis MN 55454, USA

**Keywords:** physical activity, social support, environmental resources, perceived competence

## Abstract

**Background:**

Most Americans are not active at recommended levels. Adolescence is a developmental period when physical activity (PA) decreases markedly.

**Methods:**

This study investigates whether access to environmental PA resources moderates the relationship between psychosocial resources (social support and perceived competence) and PA among 192 adolescents.

**Results:**

Environmental access to PA resources (determined via GIS-based assessment of the number of gyms, schools, trails, parks and athletic fields within 0.5 miles of each participant's home) moderated the association between social support and PA; among adolescents with high levels of environmental resources, greater social support was associated with students participating in a greater number of sports in school, whereas no such relationship emerged among adolescents with low environmental resources.

**Conclusions:**

PA-promotion interventions should aim to enhance *both *social and environmental resources; targeting either one alone may be insufficient.

## Background

Physical activity (PA) is linked to a wide range of health benefits including enhanced cognitive function, attention, and energy levels, improved body composition (i.e. less body fat, stronger bones, skeletal muscle and cardiovascular muscle), and prevention of cardiovascular disease, diabetes, cancer, hypertension, obesity, depression, osteoporosis, and premature death [1-3]. Conversely, physical inactivity is associated with increased morbidity, even among young people [4-6]. Despite the substantial evidence of PA's health benefits, adolescence is a time period when many individuals transition from activity to inactivity [7].

Investigations of the drop-off of PA that typically occurs during adolescence have identified psychological, social, and environmental factors as influential [see [8] for review]. Two psychosocial variables consistently related to PA among young people are social support [9-11] and perceived physical competence. Support from family members and friends is consistently and positively associated with PA [8,12-14]. Recent research also has suggested a positive link between PA and perceived physical competence [8,13,15-17], although several early studies reported no association between perceived physical competence and PA [see [18] for review]. A substantial body of literature also supports the link between environmental factors and adolescent PA [e.g. [19-23]]. A recent review reports that access to equipment and facilities is a primary factor related to adolescent PA [24]. Adolescents who reside within close proximity of amenities like schools and parks tend to be more physically active than adolescents who live farther away from these resources [24].

Health behavior theories emphasizing the interrelationships between multiple factors, such as Social Ecological Theory [25], suggest that determinants at different levels (e.g. psychological, social, and environmental) interact with one another to influence health behaviors like PA. Research examining interactions between environmental and individual-level factors as they relate to PA is in its early stages [see [26]] but this type of research suggests that environmental features do not impact behavior equally across individuals. For example, perceived safety from traffic was only associated with active transportation among youth with low self-efficacy [27], and expanded access to outdoor individual sports facilities increased physical activity only among adults who had low self-efficacy for exercising [28]. These findings are consistent with Social Ecological Theory and suggest that the physical environment creates a context within which individual choices may be exercised; thus, the environment may moderate the relationship between psychosocial factors and PA.

The present study builds on the existing research by investigating whether environmental and psychosocial resources interact to influence PA behavior in concert. Specifically, this study seeks to determine whether environmental access to resources that facilitate PA moderates the relationship between psychosocial resources (i.e. social support and perceived competence) and PA behavior among adolescents. This study extends the literature by employing an objective measure of environmental resources for PA (i.e. GIS) as well as multiple measures of PA, including an objective measure (i.e. accelerometer). Consistent with prior research, we hypothesized that perceived physical competence, social support for PA, and environmental PA resources would be directly associated with PA. It was also hypothesized that access to environmental resources would moderate the relationship between psychosocial factors and PA because for any given level of psychosocial resources, those with easier access to environmental resources face fewer barriers to being active. Thus, the highest levels of PA were anticipated among those adolescents with high levels of both psychosocial and environmental resources for PA.

## Methods

### Participants

Participants were 192 adolescents (mean age ± S.D. = 14.79 ± 0.46 years; 55% male) who enrolled in a larger, laboratory-based study assessing PA, psychosocial constructs, and fitness [see [29] for additional information about larger study]. Participants were recruited over four consecutive summers (2005-2008), with approximately fifty adolescents per summer. Participants were recruited via fliers and class presentations at two Southern California public high schools similar in size, demographic characteristics, academic achievement, and PA facilities available for student use. Adolescents and their parents/guardians provided written informed consent to the procedures before participating. All procedures were approved by the University of California Irvine's Institutional Review Board.

### Procedure

During the summer, participants came to the University's General Clinical Research Center (GCRC) where height and weight were measured and participants completed a series of questionnaires including multiple measures of PA. Participants were provided with Actigraph^® ^accelerometers to wear for 7 days to assess PA outside of the clinic.

### Measures

#### Perceived Physical Competence

Perceived physical competence - the belief in one's own athletic/exercise proficiency - was assessed via the "competence" subscale of the Intrinsic Motivation Inventory [IMI; [30]]. This subscale includes 6 items on which respondents rate statements such as "I think I am pretty good at exercising" on a 7 point Likert scale: 1 (not at all true) to 7 (very true). The scale showed good internal consistency, with a Cronbach's alpha of 0.87 in this sample. To create the composite measure used in analyses, the responses to the 6 items were averaged; the range of values for this variable among these participants was 1.5 to 7, and the mean was 5.3. Two participants did not provide responses to this questionnaire, but among the other 190 participants there were no missing data.

#### Social Support for PA

Social support for exercise includes the provision of either emotional encouragement or tangible assistance to further a PA-related end. Two scales [11] were used to assess social support for PA: one measured friend support and one measured family support. Each five-item scale asked respondents about their PA in a typical week (e.g. how often friends and family members participated in PA with them or encouraged them to participate in PA). Response options ranged from 1 (never) to 5 (every day). The family and friend support measures showed acceptable internal consistency in this sample, with Cronbach's alphas of 0.77 and 0.76, respectively. A combined support variable was created by averaging scores of all 10 family and friend support items; this variable is labeled "social support for PA" and was utilized in analyses due to the strong [31] positive correlation (*r *= 0.54) between family support for PA and friend support for PA; also, analyses utilizing the measures separately demonstrated the same relationships with PA in nearly all cases. Among these adolescents, the range of scores on this composite variable was 1.3 to 4.7. Two participants did not provide responses to this questionnaire, but among the other 190 participants there were no missing data.

#### Environmental Resources for PA

Geographic Information Systems (GIS) mapping software (ArcGIS, ESRI, Redlands CA) was used to assess whether adolescents lived within a neighborhood distance [0.5 mile; [32]] of several environmental features capable of influencing PA. The environmental features examined have all been linked to PA in recent research [33-37] and included: paved, off-road bicycle trails; gyms; parks (traditional open green space parks and skate parks); athletic fields (baseball diamonds, soccer fields, etc.); and the adolescents' schools. A composite environmental resources score was calculated for each participant by summing the number of these resources available within a 0.5 mile radius of his or her home. When one of these resources was located within the confines of another (e.g. a baseball diamond or pedestrian trial in a park; an athletic field or gym at a school), the space was only counted once. Scores on this variable ranged from 0 to 6; mean, median, and modal values were all approximately or exactly 2.

#### Physical Activity

Four assessments of PA were obtained: 1) Moderate to Vigorous Physical Activity (MVPA) via Actigraph^® ^accelerometer; 2) 3-Day Physical Activity Recall (3DPAR); 3) self-reported participation in school sports; 4) self-reported participation in out-of-school sports.

##### Actigraph^®^

Accelerometers are motion sensors that record magnitude of acceleration, allowing for the determination of PA intensity as well as quantity. Accelerometers used in the present study were Actigraph^® ^waist units (model 7164, ActiGraph, Pensacola, FL), worn by the participants for a minimum of 4 and a maximum of 7 days, during all waking hours, except when swimming or bathing, on a belt with the unit positioned at the left hip. A review of the objective PA measurement literature [38] found that the accelerometers used in the present study are the only commercially available devices to correlate with the gold-standard doubly labeled water technique for measuring energy expenditure. Accelerometers have been validated for PA measurement in both laboratory and field settings [see [39]] and provide minute-by-minute numerical descriptions of the activity in which the individual wearing the unit is engaging. Raw data are presented in counts per minute which, in this study, were converted to light, moderate, and hard PA based on validated [40] cutpoints [light activity: < 1952 counts; moderate: 1952 - 5724; hard: > 5724]). Using these levels, total MVPA was summed over the course of the day and averaged across all complete days of wear (defined as at least 8 hours of Actigraph^® ^use). Among these adolescents, the range of accelerometer-derived MVPA values was 6.3 to 176.9 minutes per day.

##### Self-reported PA

Self-reported PA was assessed via three questionnaires, which measured: 1) school sports participation, (i.e. adolescents indicated whether or not they played each of 14 school sports or others not listed, which elicited a range from 0 to 5 school sports in this sample); 2) sports participation outside of school, (adolescents indicated whether or not they played each of 14 non-school sports or others not listed, which produced a range of 0 to 6 sports in this sample); and 3) the 3-Day Physical Activity Recall (3DPAR) self-report assessment, validated by Motl, Dishman, Dowda, and Pate [41]. The 3DPAR requires participants to report their activities for each 30-minute block of time between 7:00 am and 11:30 pm on the three previous days. Activities were converted into metabolic equivalents (METS) using the compendium published by Ainsworth et al. [[42], updated [43]], from which average daily minutes of MVPA (≥3 METS) was calculated. The range of 3DPAR-derived daily MVPA values among these adolescents was 0 to 540 minutes per day.

#### Parent PA Behavior and Perceptions

Parental PA behavior and parental perception of benefits associated with child's PA were included as covariates to control for the possibility that a relationship between environmental resources for PA and adolescent PA might represent the influence of physically active parents self-selecting neighborhood environments with greater access to PA opportunities. This selection bias, coupled with active parents' ability to transmit their preference for PA via both nature and nurture [44,45], could create a spurious PA-environment relationship among adolescents; thus, parental PA and perceptions were statistically controlled in all analyses.

##### Parental PA

Adolescents were asked: "How often does each of your parents (or legal guardians) participate in at least 30 minutes of exercise that is hard enough to increase breathing and heart rate?" Responses ranged from 1 (rarely) to 4 (at least 5 times per week) and were provided for both parents/guardians. Ranges were 1 to 4 for both parents in this sample.

##### Parental perceptions of benefits attained through child's PA

One parent/guardian completed a questionnaire that assessed perceived benefits of PA for their child. Parents were asked to complete the stem "If my child participates in regular physical activity or sports, then: " by endorsing how strongly (from 1 [strongly disagree] to 5 [strongly agree]) they agreed with 14 statements describing potential psychological, social, and physical health benefits of PA (e.g., "S/he will meet new people," "S/he will feel less tension and stress.") This scale was modified from a tool used with college students [46] and showed good internal consistency, with a Cronbach's alpha of 0.91 in this sample. To create the composite measure used in analyses, the responses to the 14 items were summed; the range of values for this variable among these participants was 14 to 70.

#### Sociodemographic Covariates

Participant age, gender, and race were self-reported. Socioeconomic status was determined via median household income for the Census block group (2000 Census) in which the participant lived.

### Data Analyses

Primary analyses were three sets of ordinary least squares regression analyses exploring the relationships among the following constructs: psychosocial resources for PA, environmental resources for PA, and PA itself. All analyses controlled for participant age, gender, race, parental PA, parental PA perceptions, socioeconomic status (SES) and body mass index (BMI) percentile, based on known associations with psychosocial and environmental predictors. Analyses incorporating Actigraph^® ^data utilized only those participants (n = 130) who reported at least 8 hours of wear time on at least 4 days, including 2 weekend days and also included the number of days with complete data as a covariate.

The first set of regression analyses examined the psychosocial variables (social support and perceived physical competence) individually as predictors of each measure of PA [MVPA(Actigraph^®^), MVPA(3DPAR), school sports, and out-of-school sports].

The second set of regression analyses examined environmental access to PA resources as a predictor of each measure of PA. The third set of analyses examined the interaction between psychosocial and environmental resources for PA as a predictor of PA to investigate whether environmental access to PA resources moderated the relationships between psychosocial resources and PA. The environmental and psychosocial variables were centered prior to computing the multiplicative interaction terms. Each interaction term was tested as a predictor of each PA variable in models including both of the main effects comprising the interaction term, again controlling for sociodemographic and parental variables, as well as BMI percentile.

To investigate the nature of significant interactions, regression lines were plotted representing groups high (one SD above the mean) versus low (one SD below the mean) on environmental resources at high and low levels of the psychosocial variable. The simple slopes of the two lines (high vs. low access) were then calculated and tested for statistical significance (i.e., whether the slopes were significantly different from zero; see [47].)

## Results

### Descriptive Statistics

Participant characteristics are provided in Table 1 for all sociodemographic, psychosocial, PA, and parent variables involved in analyses. There were no statistically significant differences for any of the predictor or control variables between the subset of participants with complete Actigraph^® ^data and those with incomplete Actigraph^® ^data. Thus, analyses of all four measures of PA appear to reflect the characteristics of the complete sample. The most commonly played school sports were swimming/diving, track/field, and football. The sports most commonly played outside of school were swimming/diving, soccer, and tennis. Table 2 provides a complete listing of sports played in- and out-of-school for both males and females.

**Table 1:** Sample Descriptive Characteristics

	Total Sample *n *= 192	**Participants with Complete**^**a **^**Actigraph**^® ^**Data *n *= 130**
	***N *(%)**	***N *(%)**

Male	105 (54.7%)	69 (53.5%)
Race		
African American	1 (0.5%)	0 (0%)
Asian/Asian American	17 (8.9%)	12 (9.3%)
White	130 (67.7%)	87 (67.4%)
Other Race	26 (13.5%)	19 (14.7%)
Decline to State	18 (9.4%)	11 (8.5%)
School Sports		
0 sports:	67 (34.9%)	49 (38.0%)
1 sport:	69 (35.9%)	41 (31.8%)
>1 sport:	56 (29.2%)	39 (30.2%)
Out-of-School Sports		
0 sports:	40 (20.8%)	31 (24.0%)
1 sport:	78 (40.6%)	49 (38.0%)
>1 sport:	74 (38.6%)	49 (38.0%)

	*Mean (SD)*	*Mean (SD)*

Age (Years)	14.79 (0.46)	14.81 (0.47)
Household Income by Block	$88,774.50	$86,755.05
Group (U.S. Dollars, 1999)	($28,881.82)	($27,306.47)
Perceived Physical Competence	5.27 (1.21)	5.21 (1.18)
Social Support for PA		
Combined Support:	3.14 (0.73)	3.12 (0.76)
Family Support:	3.03 (0.89)	3.01 (0.93)
Friend Support:	3.25 (0.76)	3.23 (0.77)
Environmental PA resources	2.20 (1.45)	2.22 (1.48)
3DPAR (MVPA min/day)	211.09 (115.81)	199.38 (119.59)
Actigraph^® ^(MVPA min/day)	NA^a^	44.15 (27.06)
BMI	21.99 (3.77)	21.93 (3.57)
BMI Percentile	60.38 (27.34)	60.66 (27.58)
Parental PA (mother)	2.33 (1.06)	2.40 (1.09)
Parental PA (father)	2.34 (1.07)	2.36 (1.08)
Parental value of PA	62.26 (7.76)	62.00 (8.42)

^a^Actigraph^® ^data were considered complete if data were recorded for ≥8 hours on ≥4 days, including 2 weekend days.

**Table 2:** School Sports and Out-of-School Sports Participation by Gender

Sport	School Sports Participants		Out-of-School Sports Participants	
	
	Male	Female	Male	Female
Aerobics	0	0	4	7
Base/Softball	4	3	9	4
Basketball	11	6	14	9
Bicycling	0	0	1	3
Cheerleading	0	5	0	3
Color guard	0	2	0	1
Cross country	5	7	0	0
Dancing	0	0	0	17
Football	20	0	10	0
Golf	4	3	6	2
Gymnastics	0	0	2	6
Hockey	2	0	3	0
Horseback riding	0	0	0	2
Lacrosse	5	3	3	2
Martial arts	0	0	8	8
Paintball	0	0	1	0
Pilates	0	1	0	2
Rugby	1	0	0	0
Running	0	0	9	6
Skating	0	0	0	0
Skiing	0	0	5	4
Soccer	7	11	16	20
Surfing	0	0	0	1
Swimming/Diving	12	14	20	24
Tennis	2	6	11	13
Track and field	13	11	0	0
Volleyball	6	5	4	5
Water polo	11	7	8	4
Weightlifting	1	0	0	0
Wrestling	10	1	3	1

### Psychosocial Variables Predicting Physical Activity

Regression analyses examining the psychosocial variables (perceived competence and social support for PA) in relation to PA revealed that both of the psychosocial variables predicted all four measures of PA in the expected, positive direction, and accounted for between 2.6% and 17.7% of the variance in physical activity (see Table 3). All relationships were statistically significant at the p < 0.05 level.

**Table 3:** Psychosocial and Environmental Predictors of Physical Activity^a ^(n = 190^b^)

	**MVPA (Actigraph**^®^**)**^**c**^		MVPA (3DPAR)		School Sports		Out-of-School Sports	
	***b*(*se*)**	**Semi-partial corr**^**2 d**^	***b*(*se*)**	**Semi-partial corr**^**2 d**^	***b*(*se*)**	**Semi-partial corr**^**2 d**^	***b*(*se*)**	**Semi-partial corr**^**2 d**^

Perceived Competence	0.043* (0.020)	0.030	1.991** (0.745)	0.037	0.303*** (0.045)	0.177	0.183*** (0.045)	0.073
Social Support	0.063* (0.032)	0.027	2.639* (1.186)	0.026	0.474*** (0.072)	0.173	0.313*** (0.071)	0.086
Environmental Resources	0.096~ (0.052)	0.023	0.954 (2.006)	0.001	0.234~ (0.133)	0.015	0.046 (0.125)	0.001

*~p *< 0.08; *p *< 0.05*; *p *< 0.01**; *p *< 0.001***

^a ^Regression equations predicting PA included gender, age, race, BMI percent, household income, parental PA and parental value of PA as covariates.

^b ^Two participants did not provide any responses to the Perceived Competence and Social Support questionnaires, for a total sample size of 190.

^c^Analyses using Actigraph^® ^data were performed on a subsample of adolescents (n = 130) who had ≥4 complete days of data (≥8 hours of wear-time), including 2 weekend days. These analyses also included the number of complete days as a covariate.

^d ^Squared semi-partial correlations represent the proportion of the variance in MVPA(Actigraph^®^), MVPA(3DPAR), school sports participation, and out-of-school sports participation explained by perceived competence and social support for PA.

### Environmental Access Predicting Physical Activity

Regression analyses examining GIS-measured environmental access to PA resources (gyms, schools, trails, parks and athletic fields) in relation to PA indicated that environmental access was not a statistically significant predictor at the p < 0.05 level of any of the four measures of PA (see Table 3). Environmental access predicted school sports participation and accelerometer-determined MVPA at trend levels (p < 0.08).

### Environment as Moderator of Relationship between Psychosocial Resources and PA

**Figure 1:**
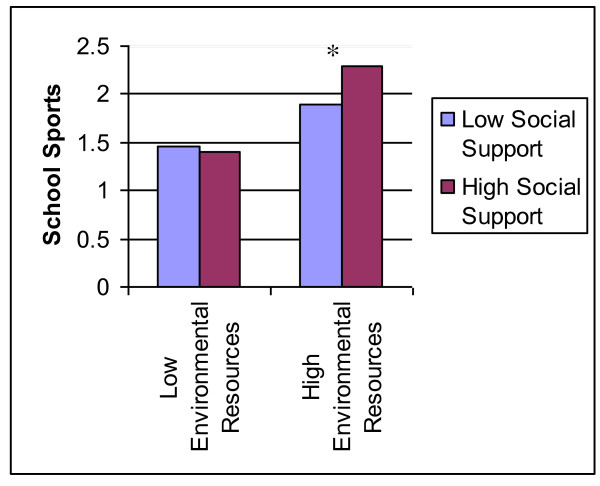
**Predicted School Sports Participation for Adolescents with High vs. Low Social Support by Environmental Resources**. *p < 0.05 Analyses were conducted using continuous variables, which were centered on their mean values; predicted levels of school sports participation were plotted for both high and low environmental resources for PA for subjects scoring 1 standard deviation above and below the mean level of social support for PA for illustrative purposes.

Tested next was whether access to environmental PA resources moderated the observed relationships between psychosocial resources and PA. The interaction between environmental resources and mean social support for PA was a statistically significant predictor of school sports participation (*b = *0.35, *SE *= 0.16, *p *< 0.05). Plotting this interaction (see Figure 1) revealed the nature of the moderation: among adolescents with a relatively high level of environmental resources, greater social support was associated with higher levels of sports participation (*b *= 0.49, *SD *= 0.18, *p *< 0.01). This relationship was absent among adolescents with relatively low environmental resources (*b *= -0.03, *SD *= 0.15, *p *= 0.82). Environmental PA resources did not moderate any of the other relationships between a psychosocial measure and a PA measure. Post-hoc analyses revealed statistically significant correlations between participation in school sports and measures of both aerobic fitness and body composition. Specifically, there were moderately sized correlations between school sports participation and both cycle ergometer-derived VO_2_peak (r = 0.36) and Dual Energy X-ray Absorptiometry (DEXA; r = -0.26).

## Discussion

This study examined the relationship between psychosocial resources (perceived physical competence and social support for PA), environmental resources for PA, and measures of PA. Consistent with hypotheses and previous research, higher levels of social support and perceived competence were associated with higher levels of PA (assessed via in-school and out-of-school sports participation, accelerometry, and self-reported PA recall). Interestingly, environmental resources did not display a direct relationship with any of the PA variables in this study. However, there was a significant interaction between environmental access and social support. Specifically, among adolescents with high levels of environmental resources, greater social support was associated with students participating in a greater number of sports in school, whereas no such relationship emerged among adolescents with low environmental resources.

Evidence that adolescents with high levels of *both *social support and environmental PA resources participated in the most school sports, whereas adolescents with low levels of one or both of these variables were significantly less likely to participate in school sports has important implications. This finding supports the calls by Leonard Epstein [48] and others [see [24,49]] for interventions aimed at increasing PA that address both environmental influences on PA and mobilization of support for PA from members of participants' social support networks. The fact that adolescents with high levels of either environmental resources *or *social support were no more active than adolescents with low levels of both suggests that social support and environmental resources may each be necessary but not sufficient conditions for promoting adolescent PA. Thus, interventionists may see improved success in promoting PA by addressing the dual goals of increasing social support for PA and increasing access to PA opportunities.

One surprising result was that the relationship between perceived competence and PA was not moderated by access to environmental PA resources. Adolescents with higher levels of perceived physical competence showed higher levels of PA than adolescents with lower levels of perceived competence whether they had access to environmental resources or not. One possible explanation is that individuals with high perceived physical competence may not see environmental deficits as insurmountable roadblocks and may be more willing to go out of their way to be active. This result was unexpected given the recent reports of interactions between environmental variables and self-efficacy, a construct similar to perceived competence, in relation to PA [27,28].

However, the present study differed from these earlier studies in important ways beyond examining competence as opposed to self-efficacy. The current study objectively measured the environment in the United States, whereas these other studies measured the environment subjectively, and examined residents of Australia and Belgium. It is possible that these differences and others may help explain the differing results. It is also worth noting that the sample size of the present study is substantially smaller than the samples studied by Cerin and colleagues [28] and Deforche and colleagues [27], and that the relatively smaller sample size may help explain why the present study did not produce statistically significant links between the environment and PA as reported in these other studies, despite comparable effect sizes across all 3 studies.

Another surprising finding was that although all four measures of PA had strong positive relationships with both psychosocial measures, only one of these relationships (i.e. social support and school sports participation) was moderated by environmental resources. It is possible that school sports participation is a unique and important predictor. Indeed, research suggests that involvement in organized sports as a child and adolescent increases the likelihood for a physically active lifestyle into adulthood [50]. It is also possible that idiosyncrasies of each of the other PA assessments, discussed below, could make school sports participation the most representative measure of PA among these adolescents and could help explain why the hypothesized moderation was seen for school sports participation but not for any of the other PA measures used in the present study.

The self-reporting involved in the 3DPAR may have been subject to recall bias or inflated reporting of time spent engaging in PA. Adolescents self-reported about 3 hours *more *MVPA per day on the 3DPAR than was recorded via accelerometry (see Table 1). Additionally, the 3DPAR and accelerometer data reflect only a small slice of time in the lives of these adolescents, which may not be highly representative of their year-round PA. Further, the outside-of-school-sports questionnaire elicited a range of responses (e.g. paintball, surfing, horseback riding) that likely varied a great deal more in terms of intensity and frequency than did participation in a school sport.

It is also worth noting that the 3DPAR and accelerometer data reflect PA patterns during the summer, which may differ from year-round PA behavior. Summer in Southern California is marked by high beach traffic, and it is possible that these teens were traveling to beaches for some of their PA (e.g. surfing, swimming) rather than using environmental resources nearer to their homes. Further research could investigate this possibility by objectively recording (e.g. via Global Positioning System; GPS) physical activity location. Participation in water sports has additional ramifications for the data collected by the accelerometers, which were not waterproof. Many participants reported removing the Actigraph^® ^during times that they were active, thus affecting the representativeness of the accelerometer data. However, analyses were also run using an accelerometer variable that adjusted for missing data based on participant report of when the accelerometers were removed to participate in water sports, and the results were not significantly different from those reported here.

Taken together, these factors may help explain why school sports participation (which reflects PA behavior during the majority of the year) was the PA measure in this study that behaved as hypothesized whereas the other PA measures did not; however, the measure of school sports used here was also not without flaws. The measures of sports participation did not gauge the frequency or intensity of PA and thus were not directly translatable to minutes of MVPA to compare with Actigraph^® ^and 3DPAR data. Nor did these measures distinguish between training activities for a sport (e.g. weight-lifting) and playing the sport itself, so participants may have inconsistently categorized their-sport related training as part of the sport or as another activity entirely. However, it should be noted that there was objective biological evidence suggesting that those adolescents participating in more school sports were, in fact, more active than those who participated in fewer or no school sports; specifically, participation in school sports was correlated with both physical fitness (ergometer-determined VO_2_peak) and body composition (DEXA-determined percent body fat).

The present study provides insight into the ways in which environmental and psychosocial factors can impact adolescent PA, but there are limitations to consider beyond the possible lack of year-round representativeness of some of the PA measures. First, the median household income for the participants in this study was $87,133 (range: $41,591 to $182,300), which is higher than the national median household income ($51,425) as well as the median for California ($61,154); however it is closer to the median for Orange County, where the participants resided ($75,176) [51]. In addition, the racial composition of this sample does not mirror the racial distribution across the U.S. (although the percentage of Caucasian participants in this study [68%] was not drastically dissimilar from the percentage of Caucasians living in California [61%], where the data were collected, or from the national average [74%; [51]]).

These demographic characteristics limit the generalizability of these results to other groups of adolescents; however, as reported elsewhere [52], these adolescents are quite representative of the American adolescent population in other important ways (e.g. body composition and cardiovascular fitness). It should be noted that SES was assessed at the aggregate level (i.e. each participant's SES was determined by the median household income in his or her Census block group), and likely differed from any individual participant's family income. However, there was a moderate correlation between self-reported income and median household income (r = 0.46, p < 0.01) among the final cohort of participants, from whom individual-level income information was collected.

Parent PA was reported by the child and may not have been an accurate representation of parental PA. In addition, although parental *perceptions *of PA benefits for their child were provided by a parent, these responses were only collected from one parent per participant, and thus may not reflect the perceptions of both parents. It is important to recognize that youth PA may relate to perceptions held by both parents as well as other unassessed family variables like living situation and family structure [53]. Future research might benefit from assessing these family variables as well as from collecting data from two parents when possible to evaluate additional questions regarding the associations between PA and parent-child relationships (e.g. do PA-related behaviors/attitudes of the same-sex parent have a stronger association with youth PA than those of the opposite-sex parent?). These questions could not be effectively addressed in the present research as only one parent completed a questionnaire per adolescent participant, and only 17.7% (n = 34) of parental respondents were male.

In addition, PA and psychosocial data were collected from 4 cohorts of adolescents over 4 consecutive years (2005 - 2008), but environmental data were all gathered at one time (2008). It is possible that some gyms, trails, parks and fields were opened or closed during the four years that individual-level data were collected. These changes in environmental access to PA resources cannot be examined in the present data. Relatedly, only proximity was assessed in relation to environmental PA resources. Usability was not measured in any other way. It must be acknowledged that factors other than proximity (e.g. safety, attractiveness) can impact use of environmental PA amenities. Finally, in light of the present results, future research assessing social support provision related to specific types of PA (e.g. transportation to school sport activities vs. non-school sport activities) might be a fruitful line of inquiry.

## Conclusions

The present research makes significant contributions to the literature examining determinants of PA. These results underscore the importance, highlighted by Social Ecological Theory, of examining interactions between variables at different levels of influence. Social Ecological Theory emphasizes the importance of examining multiple methods and levels of analysis as well as considering human-environment interactions and the contexts in which these interactions occur. The interactive influence of environmental- and individual-level predictors on PA reported in this study provide reason to believe that a multi-level, interactive model like that posited by Social Ecological Theory is useful in understanding human physical activity behavior.

Future research should investigate interactions between additional PA-related psychosocial (e.g. intentions, sensation seeking) and environmental variables (e.g. perceived access to PA resources, environmental safety) as they relate to PA. Effective promotion of PA has the capacity to significantly enhance public health; an improved understanding of the interrelationships between adolescent PA determinants and outcomes will help to inform researchers and policymakers in designing more inclusive and effective interventions among this important population. The results of the present study (i.e. that higher levels of PA are seen only among those adolescents with *both *environmental resources and social support; either type of resources on its own is insufficient) suggest that existing intervention strategies (e.g. support groups) may not be effective on their own. Provision of environmental resources to facilitate PA may enhance the effectiveness of activity-promotion programs. Thus, future activity promotion efforts may benefit from collaboration between intervention designers and urban planners, and future investigations of determinants of health behaviors like PA may benefit from considering the multiple and interactive factors that contribute to behavior.

## List of abbreviations

3DPAR: 3 Day Physical Activity Recall; BMI: Body Mass Index; GIS: Geographic Information Systems; MVPA: Moderate-to-Vigorous Physical Activity; PA: Physical Activity; SD: Standard Deviation; SE: Standard Error; SES: Socioeconomic Status;

## Declaration of Competing interests

The authors declare that they have no competing interests.

## Authors' contributions

DG conceived of the research question addressed in this paper, conducted the statistical analyses and drafted the manuscript. MS conceived of the larger study of which this was a part, participated in the design and coordination of the study and assisted with the writing and revising of the manuscript. SD assisted with the writing and revising of the manuscript. All authors read and approved the final manuscript.

## References

[B1] Haskell WL Lee IM Pate RR Powell KE Blair SN Franklin BA Macera CA Heath GW Thompson PD Bauman A Physical activity and public health: updated recommendation for adults from the American College of Sports Medicine and the American Heart Association Med Sci Sports Exerc 2007 39 1423 1434 10.1249/mss.0b013e3180616b27 17762377

[B2] Hillman CH Erickson KI Kramer AF Be smart, exercise your heart: exercise effects on brain and cognition Nat Rev Neurosci 2008 9 58 65 10.1038/nrn2298 18094706

[B3] Warburton DE Nicol CW Bredin SS Health benefits of physical activity: the evidence CMAJ 2006 174 801 809 16534088 10.1503/cmaj.051351 PMC1402378

[B4] Daniels SR Arnett DK Eckel RH Gidding SS Hayman LL Kumanyika S Robinson TN Scott BJ St Jeor S Williams CL Overweight in children and adolescents: pathophysiology, consequences, prevention, and treatment Circulation 2005 111 1999 2012 10.1161/01.CIR.0000161369.71722.10 15837955

[B5] Krishnamoorthy J Hart C Jelalian E The epidemic of childhood obesity: review of research and implications for public policy Social Policy Report 2006 20

[B6] Rowland TW Promoting physical activity for children's health: rationale and strategies Sports Med 2007 37 929 936 10.2165/00007256-200737110-00001 17953464

[B7] Nader PR Bradley RH Houts RM McRitchie SL O'Brien M Moderate-to-vigorous physical activity from ages 9 to 15 years JAMA 2008 300 295 305 10.1001/jama.300.3.295 18632544

[B8] Sallis JF Prochaska JJ Taylor WC A review of correlates of physical activity of children and adolescents Med Sci Sports Exerc 2000 32 963 975 10.1097/00005768-200005000-00014 10795788

[B9] Motl RW Dishman RK Saunders RP Dowda M Pate RR Perceptions of physical and social environment variables and self-efficacy as correlates of self-reported physical activity among adolescent girls J Pediatr Psychol 2007 32 6 12 10.1093/jpepsy/jsl001 16707779

[B10] Panter JR Jones AP van Sluijs EM Griffin SJ Attitudes, social support and environmental perceptions as predictors of active commuting behaviour in school children J Epidemiol Community Health 2010 64 41 48 10.1136/jech.2009.086918 19465403 PMC3703574

[B11] Prochaska JJ Rodgers MW Sallis JF Association of parent and peer support with adolescent physical activity Res Q Exerc Sport 2002 73 206 210 12092896 10.1080/02701367.2002.10609010

[B12] Duncan SC Duncan TE Strycker LA Sources and types of social support in youth physical activity Health Psychol 2005 24 3 10 10.1037/0278-6133.24.1.3 15631557

[B13] Neumark-Sztainer D Story M Hannan PJ Tharp T Rex J Factors associated with changes in physical activity: a cohort study of inactive adolescent girls Arch Pediatr Adolesc Med 2003 157 803 810 10.1001/archpedi.157.8.803 12912787

[B14] Van Der Horst K Paw MJ Twisk JW Van Mechelen W A brief review on correlates of physical activity and sedentariness in youth Med Sci Sports Exerc 2007 39 1241 1250 10.1249/mss.0b013e318059bf35 17762356

[B15] Barnett LM Morgan PJ van Beurden E Beard JR Perceived sports competence mediates the relationship between childhood motor skill proficiency and adolescent physical activity and fitness: a longitudinal assessment Int J Behav Nutr Phys Act 2008 5 40 10.1186/1479-5868-5-40 18687148 PMC2569960

[B16] Taylor IM Ntoumanis N Standage M Spray CM Motivational predictors of physical education students' effort, exercise intentions, and leisure-time physical activity: a multilevel linear growth analysis J Sport Exerc Psychol 2010 32 99 120 20167954 10.1123/jsep.32.1.99

[B17] Davison KK Schmalz DL Downs DS Hop, skip... no! Explaining Adolescent Girls' Disinclination for Physical Activity Ann Behav Med 2010 39 290 302 10.1007/s12160-010-9180-x 20393818 PMC5542820

[B18] Dishman RK Sallis JF Orenstein DR The determinants of physical activity and exercise Public Health Rep 1985 100 158 171 3920714 PMC1424729

[B19] Gordon-Larsen P McMurray RG Popkin BM Determinants of adolescent physical activity and inactivity patterns Pediatrics 2000 105 E83 10.1542/peds.105.6.e83 10835096

[B20] Saelens BE Sallis JF Frank LD Environmental correlates of walking and cycling: findings from the transportation, urban design, and planning literatures Ann Behav Med 2003 25 80 91 10.1207/S15324796ABM2502_03 12704009

[B21] Sallis JF Conway TL Prochaska JJ McKenzie TL Marshall SJ Brown M The association of school environments with youth physical activity Am J Public Health 2001 91 618 620 10.2105/AJPH.91.4.618 11291375 PMC1446652

[B22] Sallis JF McKenzie TL Conway TL Elder JP Prochaska JJ Brown M Zive MM Marshall SJ Alcaraz JE Environmental interventions for eating and physical activity: a randomized controlled trial in middle schools Am J Prev Med 2003 24 209 217 10.1016/S0749-3797(02)00646-3 12657338

[B23] Galvez MP Pearl M Yen IH Childhood obesity and the built environment Curr Opin Pediatr 2010 22 202 207 10.1097/MOP.0b013e328336eb6f 20090524 PMC2896907

[B24] Davison KK Lawson CT Do attributes in the physical environment influence children's physical activity? A review of the literature Int J Behav Nutr Phys Act 2006 3 19 10.1186/1479-5868-3-19 16872543 PMC1557665

[B25] Stokols D Translating social ecological theory into guidelines for community health promotion Am J Health Promot 1996 10 282 298 10159709 10.4278/0890-1171-10.4.282

[B26] Panter JR Jones A Attitudes and the environment as determinants of active travel in adults: what do and don't we know? J Phys Act Health 2010 7 551 561 20683098 10.1123/jpah.7.4.551

[B27] Deforche B Van Dyck D Verloigne M De Bourdeaudhuij I Perceived social and physical environmental correlates of physical activity in older adolescents and the moderating effect of self-efficacy Prev Med 2010 50 Suppl 1 S24 29 10.1016/j.ypmed.2009.08.017 19818363

[B28] Cerin E Vandelanotte C Leslie E Merom D Recreational facilities and leisure-time physical activity: An analysis of moderators and self-efficacy as a mediator Health Psychol 2008 27 S126 135 10.1037/0278-6133.27.2(Suppl.).S126 18377154

[B29] Schneider M Graham D Grant A King P Cooper D Regional brain activation and affective response to physical activity among healthy adolescents Biol Psychol 2009 82 246 252 10.1016/j.biopsycho.2009.08.003 19686800 PMC2767450

[B30] Ryan R Control and information in the intrapersonal sphere: An extension of cognitive evaluation theory Journal of personality and social psychology 1982 43 450 461 10.1037/0022-3514.43.3.450

[B31] Cohen J A power primer Psychol Bull 1992 112 155 159 10.1037/0033-2909.112.1.155 19565683

[B32] Kirtland KA Porter DE Addy CL Neet MJ Williams JE Sharpe PA Neff LJ Kimsey CD Jr Ainsworth BE Environmental measures of physical activity supports: perception versus reality Am J Prev Med 2003 24 323 331 10.1016/S0749-3797(03)00021-7 12726870

[B33] Fitzhugh EC Bassett DR Jr Evans MF Urban trails and physical activity: a natural experiment Am J Prev Med 2010 39 259 262 10.1016/j.amepre.2010.05.010 20709258

[B34] Carson V Kuhle S Spence JC Veugelers PJ Parents' perception of neighbourhood environment as a determinant of screen time, physical activity and active transport Can J Public Health 2010 101 124 127 20524376 10.1007/BF03404356 PMC6973633

[B35] Nelson NM Woods CB Neighborhood perceptions and active commuting to school among adolescent boys and girls J Phys Act Health 2010 7 257 266 20484765 10.1123/jpah.7.2.257

[B36] Timperio A Jeffery RW Crawford D Roberts R Giles-Corti B Ball K Neighbourhood physical activity environments and adiposity in children and mothers: a three-year longitudinal study Int J Behav Nutr Phys Act 2010 7 18 10.1186/1479-5868-7-18 20170507 PMC2847539

[B37] Coombes E Jones AP Hillsdon M The relationship of physical activity and overweight to objectively measured green space accessibility and use Soc Sci Med 2010 70 816 822 10.1016/j.socscimed.2009.11.020 20060635 PMC3759315

[B38] Plasqui G Westerterp KR Physical activity assessment with accelerometers: an evaluation against doubly labeled water Obesity (Silver Spring) 2007 15 2371 2379 10.1038/oby.2007.281 17925461

[B39] Freedson PS Miller K Objective monitoring of physical activity using motion sensors and heart rate Res Q Exerc Sport 2000 71 S21 29 10925821

[B40] Freedson PS Melanson E Sirard J Calibration of the Computer Science and Applications, Inc. accelerometer Med Sci Sports Exerc 1998 30 777 781 10.1097/00005768-199805000-00021 9588623

[B41] Motl RW Dishman RK Dowda M Pate RR Factorial validity and invariance of a self-report measure of physical activity among adolescent girls Res Q Exerc Sport 2004 75 259 271 15487290 10.1080/02701367.2004.10609159

[B42] Ainsworth BE Haskell WL Leon AS Jacobs DR Jr Montoye HJ Sallis JF Paffenbarger RS Jr Compendium of physical activities: classification of energy costs of human physical activities Med Sci Sports Exerc 1993 25 71 80 10.1249/00005768-199301000-00011 8292105

[B43] Ainsworth BE Haskell WL Whitt MC Irwin ML Swartz AM Strath SJ O'Brien WL Bassett DR Jr Schmitz KH Emplaincourt PO Compendium of physical activities: an update of activity codes and MET intensities Med Sci Sports Exerc 2000 32 S498 504 10.1097/00005768-200009001-00009 10993420

[B44] Pugliese J Tinsley B Parental socialization of child and adolescent physical activity: a meta-analysis J Fam Psychol 2007 21 331 343 10.1037/0893-3200.21.3.331 17874918

[B45] Alderman BL Benham-Deal TB Jenkins JM Change in parental influence on children's physical activity over time J Phys Act Health 2010 7 60 67 20231756 10.1123/jpah.7.1.60

[B46] Calfas K Sallis J Lovato C Campbell J Physical activity and its determinants before and after college graduation Medicine, Exercise, Nutrition, and Health 1994 3

[B47] Aiken L West S Multiple Regression: Testing and Interpreting Interactions 1991 Thousand Oaks: Sage

[B48] Epstein LH Integrating theoretical approaches to promote physical activity Am J Prev Med 1998 15 257 265 10.1016/S0749-3797(98)00083-X 9838972

[B49] Dubbert PM Physical activity and exercise: recent advances and current challenges J Consult Clin Psychol 2002 70 526 536 10.1037/0022-006X.70.3.526 12090367

[B50] Kjonniksen L Anderssen N Wold B Organized youth sport as a predictor of physical activity in adulthood Scand J Med Sci Sports 2009 19 646 654 10.1111/j.1600-0838.2008.00850.x 18694430

[B51] American Community Survey United States income in the past 12 months 2008 http://factfinder.census.gov/servlet/STTable?_bm=y&-geo_id=01000US&-qr_name=ACS_2009_5YR_G00_S1901&-ds_name=ACS_2009_5YR_G00_&-redoLog=false Last accessed on February 8, 2011 at

[B52] Schneider M Dunn A Cooper D Affect, exercise, and physical activity among healthy adolescents J Sport Exerc Psychol 2009 31 706 723 20384008 10.1123/jsep.31.6.706 PMC3531994

[B53] Bagley S Salmon J Crawford D Family structure and children's television viewing and physical activity Med Sci Sports Exerc 2006 38 5 910 918 10.1249/01.mss.0000218132.68268.f4 16672845

